# Supplementary material for: The causal relationship between the human gut microbiota and pyogenic arthritis: a Mendelian randomization study
Source: Front Cell Infect Microbiol. 2024 Nov 26;14:1452480. doi: 10.3389/fcimb.2024.1452480 (PMC11629706; doi:10.3389/fcimb.2024.1452480)
Supplement: Supplementary file 1 [file DataSheet1.zip › mendelian test/ebi-a-GCST90027847.csv_forest.pdf]

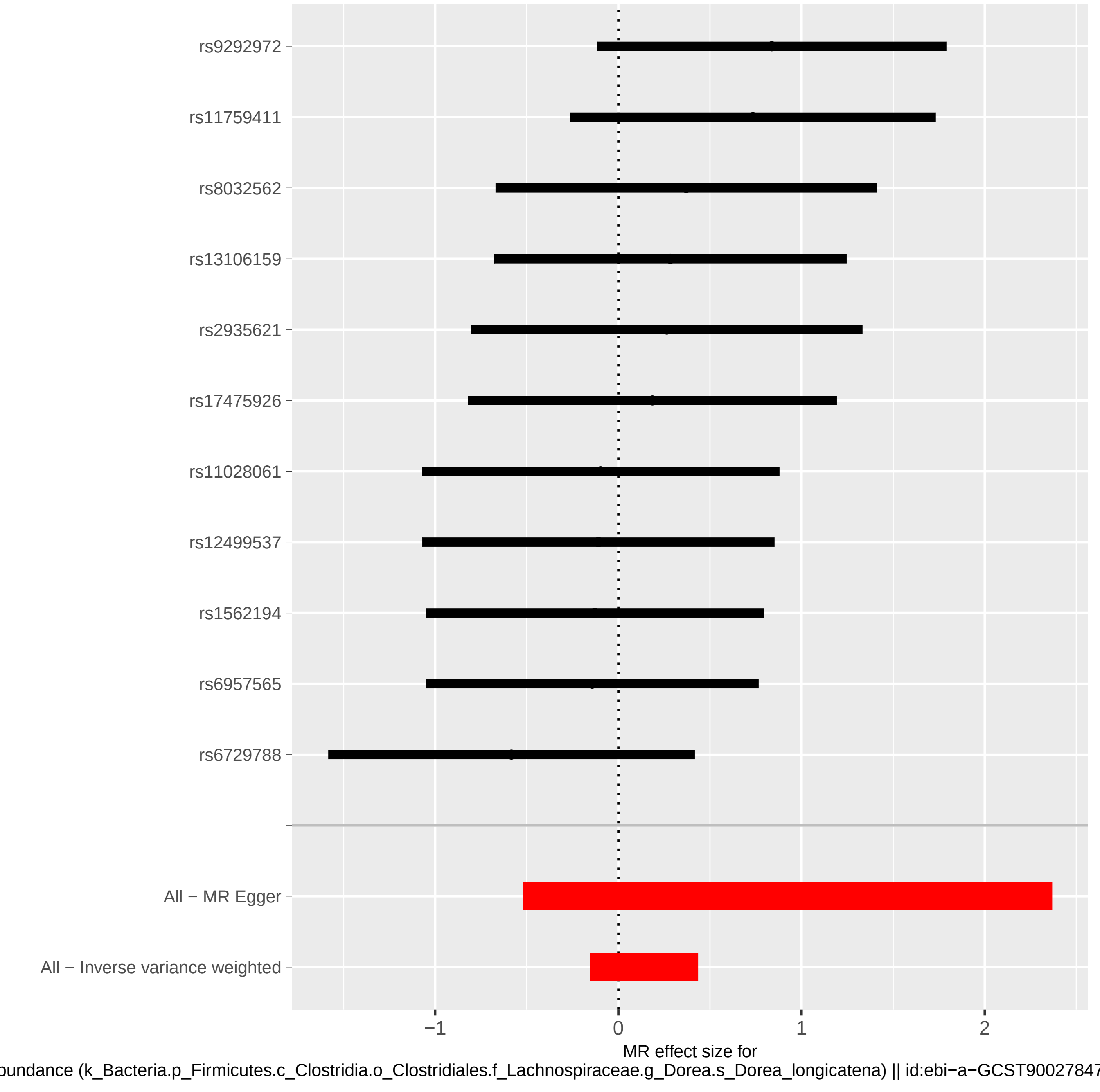
rs9292972
rs11759411
rs8032562
rs13106159
rs2935621
rs17475926
rs11028061
rs12499537
rs1562194
rs6957565
rs6729788
All − MR Egger
All − Inverse variance weighted
−1
0
1
2
MR effect size for
bundance (k_Bacteria.p_Firmicutes.c_Clostridia.o_Clostridiales.f_Lachnospiraceae.g_Dorea.s_Dorea_longicatena) || id:ebi−a−GCST90027847
